# Transcranial Doppler as a Primary Screening Tool for Detecting Right‐to‐Left Shunt in Cryptogenic Stroke Patients?

**DOI:** 10.1002/brb3.70144

**Published:** 2024-11-17

**Authors:** Payam Sasannejad, Fateme Khosravani, Alireza Ziaei Moghaddam, Mohsen Soltani Sabi, Lida Jarahi

**Affiliations:** ^1^ Department of Neurology, Faculty of Medicine Mashhad University of Medical Sciences Mashhad Iran; ^2^ Student Research Committee, Faculty of Medicine Mashhad University of Medical Sciences Mashhad Iran; ^3^ Department of Community Medicine, Faculty of Medicine Mashhad University of Medical Sciences Mashhad Iran

## Abstract

**Background:**

Cryptogenic stroke (CS), a subtype of ischemic stroke with undetermined etiology, accounts for approximately 25% of the cases. Patent foramen ovale (PFO) is an important potential cause of CS via paradoxical embolism. While transesophageal echocardiography (TEE) is the current gold standard for PFO detection, transcranial Doppler (TCD) ultrasound offers a noninvasive alternative with potential advantages in sensitivity for right‐to‐left shunt (RLS) detection. This study's main goal is to evaluate the diagnostic performance of TCD compared to TEE for PFO detection in CS patients.

**Methods:**

We prospectively enrolled 110 patients aged 18–65 years with confirmed CS from 2020 to 2024. All underwent TCD screening for RLS using a standardized protocol. Subsequently, they were categorized based on a simplified version of the Spencer Logarithmic Scale, followed by confirmatory TEE. Clinical characteristics, imaging findings, TCD results, and indications for PFO closure were analyzed.

**Results:**

The mean age of the cohort was 45 years, with 58.2% being males. TEE identified PFO in 44.5% (49/110) of subjects. TCD accurately detected RLS in 42 of the 49 PFO cases (85.7%) confirmed by TEE. For PFO detection, TCD demonstrated a sensitivity of 85.4%, specificity of 88.5%, PPV of 85.4%, and Youden's index of 0.73. Notably, of the seven PFO cases missed by TCD, none received percutaneous closure based on clinical criteria.

**Conclusions:**

TCD exhibited high diagnostic accuracy for detecting high‐risk PFO in patients with CS when compared to the gold‐standard TEE. As a noninvasive modality, TCD may serve as an effective screening tool to identify CS patients who could potentially benefit from confirmatory TEE and subsequent PFO closure intervention. The findings support the use of TCD as a screening tool to triage CS patients for confirmatory TEE and potential PFO closure.

## Graphical abstract

TCD exhibited high diagnostic sensitivity and specificity for detecting high‐risk PFO in patients with CS when compared to the gold‐standard TEE. As a noninvasive modality, TCD may serve as an effective screening tool to identify CS patients who could potentially benefit from confirmatory TEE and subsequent PFO closure intervention.

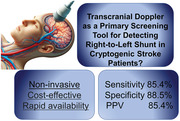

**Abbreviations**
ACA: anterior cerebral artery
ASA: atrial septal aneurysm
ASD: atrial septal defect
ASPECT: Alberta Stroke Program Early CT Score
CS: cryptogenic stroke
DW‐MRI: diffusion‐weighted magnetic resonance imaging
HITS: high‐intensity transient signals
MCA: middle cerebral artery
PASCAL: prospectively validated PFO‐associated stroke causal likelihood
PCA: posterior cerebral artery
PCASPECT: Posterior Circulating Alberta Stroke Program Early CT Score
PFO: patent foramen ovale
RLS: right‐to‐left shunt
RoPE: risk of paradoxical embolism
TCD: transcranial Doppler
TEE: transesophageal echocardiography
TIA: transient ischemic attack
VM: Valsalva maneuver

## 1 Introduction

Cryptogenic stroke (CS), a subtype of ischemic stroke with no determined etiology, accounts for 25% of ischemic stroke cases (Mohr 1988).

Recent studies have shown that a patent foramen ovale (PFO) causing a right‐to‐left shunt (RLS) is an important pathogenesis for CS due to paradoxical embolism. This occurs because PFO creates a pathway for blood clots to move from the veins to the cerebral circulation (Fonseca and Ferro 2015; Elmariah et al. 2014). CS patients with PFO demonstrate unique patterns of infarction on diffusion‐weighted imaging, distinct from those patients without a PFO. Moreover, this differentiation is further accentuated by the escalation of shunt severity (Stecco et al. 2017; He et al. 2018; Nam et al. 2019). Recent studies have found a significant reduction in recurrence risk for patients with CS and PFO after PFO closure with antiplatelet therapy. Hence, screening PFO in CS patients has become increasingly important (Mas et al. 2017; Søndergaard et al. 2017; Spencer et al. 2004).

Transesophageal echocardiography (TEE) is currently regarded as the gold standard for detecting PFO (Schneider et al. 1996). However, it has been questioned whether it has the highest sensitivity in identifying RLS (Wessler et al. 2015). In addition, the procedure is often met with low patient tolerance and necessitates profound sedation. This, in turn, restricts the patient's capacity to execute a Valsalva maneuver (VM) effectively (Spencer et al. 2004).

Transcranial Doppler (TCD) ultrasound, a noninvasive diagnostic method, exhibits a sensitivity of up to 100% in detecting RLS when compared to TEE. The accuracy of detection depends on the center's expertise, protocol, and diagnostic criteria (Sloan et al. 2004).

This noninvasive technique identifies high‐intensity transient signals (HITS) as they traverse the middle cerebral or vertebral arteries (Blersch et al. 2002; Komatsu et al. 2017; Seidel, Maps, and Gerriets 1995).

To mitigate the risk of recurrent stroke in patients with ischemic stroke associated with PFO, therapeutic interventions such as antithrombotic therapy or percutaneous device closure of the PFO are advocated in accordance with established clinical practice guidelines (Kleindorfer et al. 2021; Pristipino et al. 2019; Messé et al. 2020).

In this study, we aimed to assess the diagnostic value of TCD for the detection of PFO in patients with CS who have been confirmed with TEE.

## 2 Materials and Methods

In our study, we included patients hospitalized in Qaem Hospital's neurology department who were confirmed with stroke aged between 18 and 65 from 2020 to 2024. The imaging evaluation of these patients' infarct was based on diffusion‐weighted magnetic resonance imaging (DW‐MRI) images and was interpreted by Stroke Fellowship accompanied by an expert radiologist. We enrolled patients with cerebral, cerebellar, and brainstem infarcts. However, we specifically excluded patients with lacunar infarction from our study. The territory of lesion involvement is divided into anterior circulation (which consists of the anterior cerebral artery [ACA] and middle cerebral artery [MCA]) and posterior circulation (consisting of the posterior cerebral artery [PCA]) or multiple territories combined. That being so, Alberta Stroke Program Early CT Score (ASPECT) or Posterior Circulating Alberta Stroke Program Early CT Score (PCASPECT) for the patients' infarct is measured (Mokin et al. 2017; Puetz et al. 2008).

The patients' diagnosis was made by ruling out other possible causes of infarct. We conducted computed tomography angiography and magnetic resonance angiography of the cerebral and neck vessels. The primary objective was to exclude dissection, thrombus formation, or atherosclerosis as potential etiologies for stroke. In addition, we employed a 48‐h Holter monitoring to assess heart rhythm and exclude cardiac arrhythmias. Furthermore, transthoracic echocardiography was performed to rule out other contributory factors, such as heart failure or structural abnormalities associated with cerebral ischemia. Hypercoagulable factors, including Leiden factor V, serum homocysteine, antithrombin III, prothrombin gene mutation, lupus anticoagulant, anticardiolipin antibody, beta‐2 glycoprotein, and C and S protein levels were measured to rule out coagulopathies. After obtaining informed consent, 152 patients were enrolled in the study as having CS.

In instances where patients did not provide informed consent to participate in the study or lacked the appropriate temporal window (specifically, thickening of the temporal bone) necessary for recording HITS from the MCA, as well as cases where patients were unable to cooperate in performing the VM due to factors such as loss of consciousness or aphasia, those individuals were excluded from the study. Specifically, one patient was excluded due to an inadequate temporal window, while 41 patients were excluded because of uncooperativeness or unconsciousness. Consequently, the study was conducted with a total of 110 patients.

After taking some medical history to assess the risk of possible CS, the risk of paradoxical embolism (RoPE) score was calculated for each patient based on factors such as their age, history of hypertension, diabetes, smoking habits, previous stroke or transient ischemic attack (TIA), and any signs of cortical infarction on imaging tests (Kent et al., 2013).

**FIGURE 1:**
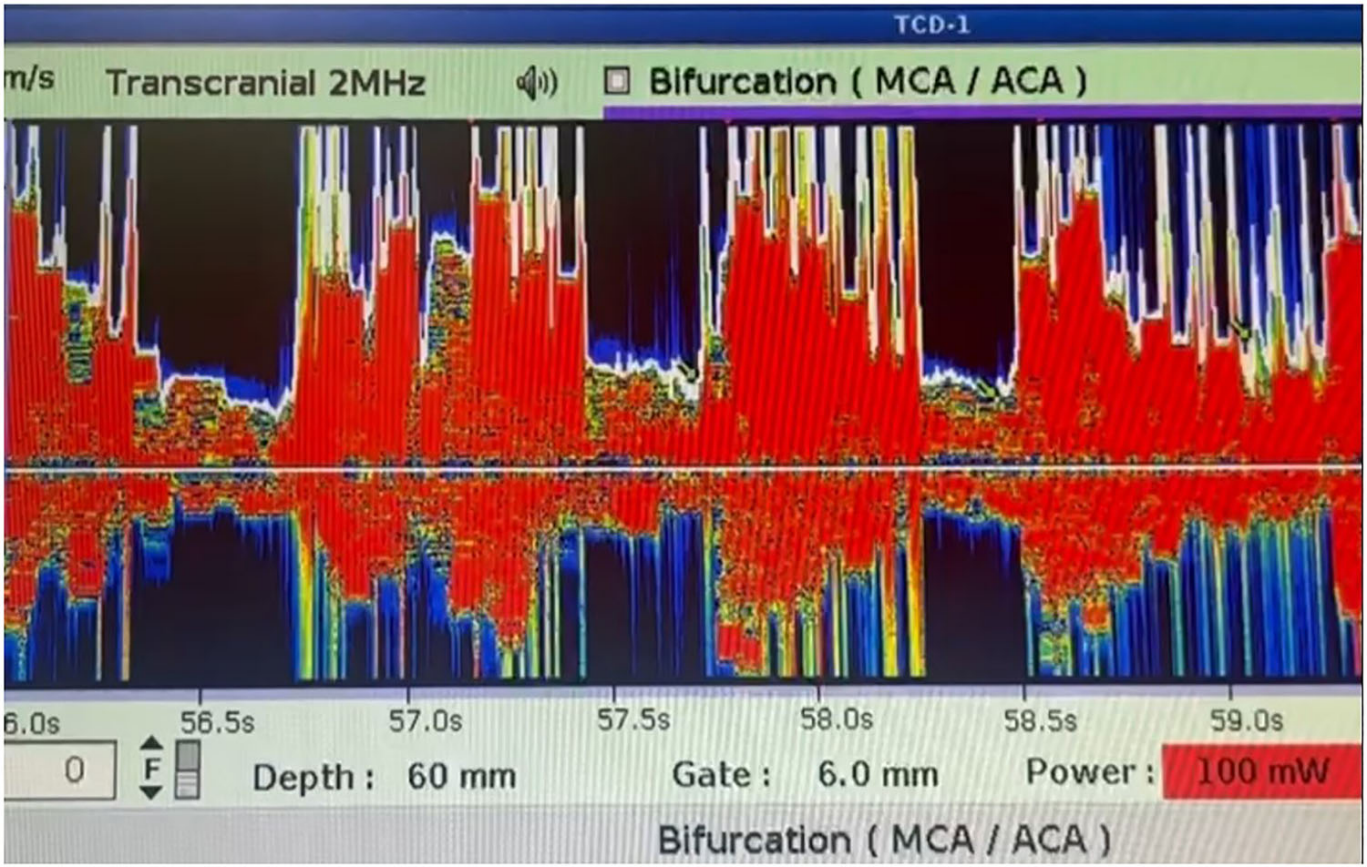
The left MCA artery was monitored through the temporal window at a depth of 48–60 mm. Grade III microbubbles were detected after the conventional VM.

During the acute phase of ischemic stroke, TCD was performed based on the standard TCD protocol (Palazzo et al. 2019) in the following manner:

The patient lies on their back, and a mixture of the patient's blood (1 cc) and air (1 cc) diluted in isotonic normal saline (8 cc) is injected into the antecubital vein of the patient. A Stroke Fellowship monitored patients using a TCD device for HITS (Figure 1), which are defined as short‐term visible and audible signals with high intensity in the Doppler flow spectrum. If no HITS are detected, the wave recording is repeated after the VM. If the result is still negative, the test is repeated after another VM. When RLS is present, foamy bubbles enter the left side through the shunt. These bubbles are recorded by an ultrasound transducer in the temporal region, which monitors the flow passing through the M1 part of the MCA artery. We considered the test positive if at least one HIT occurred within 40 seconds of the injection. The assessment of RLS magnitude involved quantifying the number of HITs detected in the MCA during the initial 40 s following bolus infusion. In addition, we employed a simplified version of the Spencer Logarithmic Scale to grade them, which consists of four visual categories: Grade 0 (no signals), Grade I (1–10 signals), Grade II (11–30 signals), and Grade ≥ III (> 30 signals) (Table 1) (Spencer et al. 2004).

**TABLE 1:** Patients' characteristics.

Characteristic or test results		*p* value
Sex, female, *n* (%)	46 (41.8%)	0.943
Smoking habit, *n* (%)	28 (25.5%)	0.056
Diabetes, *n* (%)	18 (16.4%)	0.317
Hypertension, *n* (%)	41 (37.3%)	0.224
History of CVA or TIA, *n* (%)	7 (6.4%)	0.948
Cortical infarct, %	48.05%	0.240
ASPECT, mean (SD)	8.1 (1.5)	0.348
PCASPECT, mean (SD)	8.54 (0.87)	0.943
RoPE score, mean (SD)	6.17 (1.83)	0.116
TCD results		
Grade I, %	19.51%	—
Grade II, %	36.58%	—
Grade III, %	43.90%	—
Total, % (*n*)	100 % (48)	<0.01
TEE results, positive numbers (%)	49 (44.5%)	—

All patients underwent subsequent TEE examinations following a standardized protocol (J.‐Y. Lee et al. 2010), administered by experienced echocardiography fellowships.

Finally, clinical characteristics, including demographic factors, findings of TEE and history of smoking, diabetes mellitus, hypertension, history of cerebrovascular accident (CVA) or TIA, RoPE score, ASPECT/PCASPECT, TCD findings, including grading (based on using the Spencer grading system) and indication for PFO closure, were compared between subjects. To see if there is a significant relationship with RoPe, we dichotomized the RoPE score at < 7 versus ≥ 7 (Kent et al. 2020).

In our study, we employed the prospectively validated PFO‐associated stroke causal likelihood (PASCAL) risk stratification system. This system comprises two key domains: the first pertains to high‐risk PFO features, characterized by a large shunt (defined as the passage of more than 20–30 bubbles on TEE), the presence of atrial septal aneurysm (ASA), or both. The second domain involves the RoPE score, which is further categorized into two ranges: 0–6 and 7–10 (Sposato et al. 2024). By integrating the PASCAL system with input from our interventional cardiologist, we arrived at the appropriate closure indication for these patients.

Written consent was obtained from patients before performing each step. This study's approved number is IR.MUMS.MEDICAL.REC.1401.486.

### 2.1 Statistics

SPSS, version 19.0, was used for statistical analysis. The chi‐square test or Mann–Whitney test, according to the type of variable, was used to assess the significant differences in expected frequencies between the two groups. *p* < 0.05 was considered statistically significant in a two‐sided test.

## 3 Results

A total number of 110 consecutive 18‐ to 65‐year‐old patients hospitalized for a suspected CS were screened. The average age of the enrolled patients was 45 years of whom 58.18% were men. Demographics and vascular risk factors of the 110 patients with CS are presented in Table 1.

Patients with infarct on DW‐MRI were categorized based on the territory of the infarct. Of these patients, 55.88% had infarcts in the anterior circulation (including ACA, MCA, ACA + MCA), 32.35% in the posterior circulation (PCA), and 11.76% in both circulations. For all patients, ASPECT or PCASPECT and their median are shown in Table 1 and also a graph is created to help visualize the percentages.

TEE showed the prevalence of RLS to be 44.54% (49 patients). RLS was found to be due to a PFO in all the study subjects except for one, in whom TEE showed an atrial septal defect (ASD).

No significant relationship was found between the incidence of PFO and age, sex, history of smoking, diabetes, hypertension, history of CVA or TIA, cortical infarct, RoPE score, and ASPECT/PCASPECT.

When comparing the TCD results with TEE, a significant relationship was found with a *p* < 0.01. In addition, when assessing shunt grading using the simplified Spencer Logarithmic Scale, we found that the probability of detecting RLS on TEE significantly differs across groups (Grade I, Grade II, and Grade III groups) with a *p* < 0.01. Furthermore, the probability of detecting RLS on TEE increases as the grading increases from Grade I to Grade III, with a positive coefficient of 2.43.

The sensitivity, specificity, positive predictive value, and Youden's index of TCD for diagnosis of CS patients with PFO were 85.41%, 88.75%, 85.41%, and 0.73, respectively.

Out of 48 PFO‐diagnosed patients, 12 received oral treatment and 36 had PFO closure.

In the subsequent follow‐ups of the patients, no complications related to TEE and angiography occurred.

Out of the seven cases that were not detected with TCD, no one had an indication of closure, indicating the efficiency of the test when dealing with high‐risk PFOs.

## 4 Discussion

This study demonstrated the efficacy of TCD in identifying high‐risk patients with PFO who could potentially benefit from percutaneous device closure.

TEE serves as the gold standard for evaluating the risk associated with a high‐risk PFO. TEE is capable of detecting the presence of PFO or ASD, both of which can influence the severity and dimensions of ischemic brain lesions. During TEE, the transducer is positioned in the esophagus, which poses challenges for patients with neurological deficits when attempting to perform the VM, as it is an integral part of the procedure (Katsanos et al. 2016). In addition, patients may not be able to perform VM during TEE examination due to the use of anesthetics, which can lead to poor cooperation. Therefore, TEE is more sensitive to detecting RLSs while at rest, but it cannot detect tiny shunts that can only be observed during VM (Soliman et al. 2007; Kronik, Slany, and Moesslacher 1979). In addition, TCD represents a noninvasive and economically viable approach, rendering it suitable for repeated assessments. Notably, TCD enables real‐time monitoring of cerebral blood flow, an essential capability during the VM for the detection of any potential RLS (Robba and Taccone 2019; Ji and Seoung 2023). Therefore, one may question if TEE is the ultimate RLS detection gold standard as it failed to detect shunts in some cases (Rodrigues et al. 2013).

This has led to various studies over the years, with some even finding TCD to be more sensitive than TEE in detecting RLS (Søndergaard et al. 2017; Maillet et al. 2018; Tobe et al. 2016). However, it is difficult for TCD to differentiate between pulmonary and intracardiac shunts, leading to lower specificity (Laurie et al. 2010; Lovering et al. 2008; Stickland et al. 2004). In our study, we found sensitivity and specificity for TCD to be 85.41% and 88.52%, respectively. The interesting point about our study is that among 110 patients with CS, only 7 were false negatives. More interestingly among those seven patients, none had an indication for closure. A study done recently by Park et al. supports this finding (Park et al. 2021).

Some studies suggest that TCD should be used in combination with TTE as a complementary test (Park et al. 2021; Lu et al. 2022). We recommend that TCD be employed as the primary screening test during the acute stage of stroke due to its accuracy, noninvasiveness, cost‐effectiveness, rapid availability, and safety. Should the results be positive, it is imperative to conduct a TEE to ascertain the location of the shunt at the cardiac level. TEE enables direct visualization of the atrial septum's anatomical features, facilitating confirmation of whether the shunt is attributable to a PFO or another type of ASD and whether it is accompanied by ASA or not (it is worth noting that pulmonary arteriovenous fistulas, although rare, can also give rise to shunts) (Soliman et al. 2007; Kronik, Slany, and Moesslacher 1979; Menardi, Ribeiro, and Evora 2021). Furthermore, TEE remains essential for elucidating the etiologies of embolic strokes of undetermined origin. Nonetheless, should TCD results be negative or only reveal a minimal shunt subsequent to the VM, the necessity for TEE may be obviated (Katsanos et al. 2016). Also, in some cases of TCD challenges related to temporal windows, which led to the suboptimal quality of TCD signals, impact approximately 8%–20% of the population. Consequently, performing TCD in these cases becomes unfeasible (C. H. Lee et al. 2020), necessitating the use of TEE for these patients. In our study, only one patient was excluded due to an inappropriate temporal window.

The study has some limitations. First, this is a single‑center study, so the study population is not representative of the entire CS population. Further studies with larger populations and engaging multiple centers need to be performed. Second, in this study, we only examined PFO as the only RLS for CS patients, therefore further studies need to include other possible RLS causes and cross‐examine them with both methods.

In summary, TCD is a dependable technique for identifying high‐risk PFO in individuals with CS. This method is instrumental in discerning patients who might gain from device closure and we believe it should be used as the primary screening test for the detection of PFO in patients experiencing acute cerebral ischemic events of undetermined etiology. These patients can then undergo a closure procedure after confirmation of PFO using TEE.

## Author Contributions

**Payam Sasannejad**: conceptualization, methodology, validation, supervision, project administration, software, data curation, resources, writing–original draft, visualization, writing–review and editing, investigation. **Fateme Khosravani**: conceptualization, investigation, data curation, validation, project administration, writing–review and editing, software, methodology, visualization, resources, writing–original draft, supervision. **Alireza Ziaei Moghaddam**: conceptualization, investigation, data curation, validation, supervision, project administration, writing–original draft, writing–review and editing, software, formal analysis, visualization, methodology. **Mohsen Soltani Sabi**: validation, supervision, conceptualization, methodology, resources. **Lida Jarahi**: writing–original draft, validation, formal analysis, methodology, software.

## Ethics Statement

This is reviewed and approved by the Ethics Committee of Mashhad University of Medical Sciences under the code IR.MUMS.MEDICAL.REC.1401.486. Written informed consent was obtained from all subjects for the publication of this report. This study was performed in accordance with the Declaration of Helsinki of 1964 and its later amendments.

## Conflicts of Interest

The authors declare no conflicts of interest.

### Peer Review

The peer review history for this article is available at https://publons.com/publon/10.1002/brb3.70144

## Data Availability

Additional data that support the findings of this study are available from the corresponding author upon reasonable request.

## References

[brb370144-bib-0001] Blersch, W. K. , B. M. Draganski , S. R. Holmer , et al. 2002. “Transcranial Duplex Sonography in the Detection of Patent Foramen Ovale.” Radiology 225, no. 3: 693–699. 12461247 10.1148/radiol.2253011572

[brb370144-bib-0002] Elmariah, S. , A. J. Furlan , M. Reisman , et al. 2014. “Predictors of Recurrent Events in Patients With Cryptogenic Stroke and Patent Foramen Ovale Within the CLOSURE I (Evaluation of the STARFlex Septal Closure System in Patients With a Stroke and/or Transient Ischemic Attack Due to Presumed Paradoxical Embolism Through a Patent Foramen Ovale) Trial.” JACC Cardiovascular Interventions 7: 913–920. 10.1016/j.jcin.2014.01.170. 25147037

[brb370144-bib-0003] Fonseca, A. C. , and J. M. Ferro . 2015. “Cryptogenic Stroke.” European Journal of Neurology 22: 618–623. 10.1111/ene.12673. 25597418

[brb370144-bib-0004] He, D. , Q. Shi , G. Xu , et al. 2018. “Clinical and Infarction Patterns of PFO‐Related Cryptogenic Strokes and a Prediction Model.” Annals of Clinical and Translational Neurology 5: 1323–1337. 10.1002/acn3.647. 30480027 PMC6243387

[brb370144-bib-0005] Ji, M. H. , and Y. H Seoung . 2023. “Right‐to‐Left Shunt Evaluation in Cardiac Patent Foramen Ovale Using Bubble Contrast Transcranial Color‐Coded Doppler: A Cryptogenic Stroke Case.” Healthcare 11, no. 19: 2655. 10.3390/healthcare11192655. 37830692 PMC10572775

[brb370144-bib-0006] Katsanos, A. H. , R. Bhole , A. Frogoudaki , et al. 2016. “The Value of Transesophageal Echocardiography for Embolic Strokes of Undetermined Source.” Neurology 87: 988–995. 27488602 10.1212/WNL.0000000000003063 PMC5027807

[brb370144-bib-0007] Kent, D. M. , J. L. Saver , R. Ruthazer , et al. 2020. “Risk of Paradoxical Embolism (RoPE)‐Estimated Attributable Fraction Correlates With the Benefit of Patent Foramen Ovale Closure: An Analysis of 3 Trials.” Stroke 51, no. 10: 3119–3123. 10.1161/STROKEAHA.120.029350. 32921262 PMC7831886

[brb370144-bib-0008] Kent, D. M. , R. Ruthazer , C. Weimar , et al. 2013. “An Index to Identify Stroke‐Related vs. Incidental Patent Foramen Ovale in Cryptogenic Stroke.” Neurology 81, no. 7: 619–625. 10.1212/WNL.0b013e3182a08d59. 23864310 PMC3775694

[brb370144-bib-0009] Kleindorfer, D. O. , A. Towfighi , S. Chaturvedi , et al. 2021. “2021 Guideline for the Prevention of Stroke in Patients With Stroke and Transient Ischemic Attack: A Guideline From the American Heart Association/American Stroke Association.” Stroke 52, no. 7: e364–e467. 10.1161/STR.0000000000000375. 34024117

[brb370144-bib-0010] Komatsu, T. , Y. Terasawa , A. Arai , K. Sakuta , H. Mitsumura , and Y. Iguchi . 2017. “Transcranial Color‐Coded Sonography of Vertebral Artery for Diagnosis of Right‐to‐left Shunts.” Journal of the Neurological Sciences 376: 97–101. 28431637 10.1016/j.jns.2017.03.012

[brb370144-bib-0011] Kronik, G. , J. Slany , and H. Moesslacher . 1979. “Contrast M‑Mode Echocardiography in Diagnosis of Atrial Septal Defect in Acyanotic Patients.” Circulation 59: 372–378. 759005 10.1161/01.cir.59.2.372

[brb370144-bib-0012] Laurie, S. S. , X. Yang , J. E. Elliott , et al. 2010. “Hypoxia‐Induced Intrapulmonary Arteriovenous Shunting at Rest in Healthy Humans.” Journal of Applied Physiology 109: 1072–1079. 20689088 10.1152/japplphysiol.00150.2010

[brb370144-bib-0013] Lee, C. H. , S. H. Jeon , S. J. Wang , B. S. Shin , and H. G Kang . 2020. “Factors Associated With Temporal Window Failure in Transcranial Doppler Sonography.” Neurological Sciences 41, no. 11: 3293–3299. 10.1007/s10072-020-04459-6. 32405883

[brb370144-bib-0014] Lee, J.‐Y. , J.‐K. Song , J.‐M. Song , et al. 2010. “Association Between Anatomic Features of Atrial Septal Abnormalities Obtained by Omni‐Plane Transesophageal Echocardiography and Stroke Recurrence in Cryptogenic Stroke Patients With Patent Foramen Ovale.” American Journal of Cardiology 106: 129–134. 20609660 10.1016/j.amjcard.2010.02.025

[brb370144-bib-0015] Lovering, A. T. , L. M. Romer , H. C. Haverkamp , et al. 2008. “Intrapulmonary Shunting and Pulmonary Gas Exchange During Normoxic and Hypoxic Exercise in Healthy Humans.” Journal of Applied Physiology 104: 1418–1425. 18292301 10.1152/japplphysiol.00208.2007

[brb370144-bib-0016] Lu, J. , J. Li , H. Huang , and Q. Ye . 2022. “Diagnostic Value of Micro‐Bubble Transcranial Doppler Combined With Contrast Transthoracic Echocardiography in Cryptogenic Stroke Patients With Patent Foramen Ovale.” Neurology India 70, no. 4: 1403–1406. 10.4103/0028-3886.355122. 36076635

[brb370144-bib-0017] Maillet, A. , A. Pavero , P. Salaun , et al. 2018. “Transcranial Doppler to Detect Right to Left Communication: Evaluation Versus Transesophageal Echocardiography in Real Life.” Angiology 69: 79–82. 10.1177/0003319717712356. 28583003

[brb370144-bib-0018] Mas, J. L. , G. Derumeaux , B. Guillon , et al. 2017. “Patent Foramen Ovale Closure or Anticoagulation vs. Antiplatelets After Stroke.” New England Journal of Medicine 377: 1011–1021. 10.1056/NEJMoa1705915. 28902593

[brb370144-bib-0019] Menardi, A. C. , P. J. F. Ribeiro , and P. R. B Evora . 2021. “Atrial Septal Aneurysm and Atrial Septal Defect Association—An Uncommon but Well‐ Recognized Association.” Brazilian Journal of Cardiovascular Surgery 36, no. 4: 557–560. 10.21470/1678-9741-2020-0464. 34236809 PMC8522310

[brb370144-bib-0020] Messé, S. R. , G. S. Gronseth , D. M. Kent , et al. 2020. “Practice Advisory Update Summary: Patent Foramen Ovale and Secondary Stroke Prevention: Report of the Guideline Subcommittee of the American Academy of Neurology.” Neurology 94, no. 20: 876–885. 10.1212/WNL.0000000000009443. 32350058 PMC7526671

[brb370144-bib-0021] Mohr, J. P. 1988. “Cryptogenic Stroke.” New England Journal of Medicine 318, no. 18: 1197–1198. 3362167 10.1056/NEJM198805053181810

[brb370144-bib-0022] Mokin, M. , C. T. Primiani , A. H. Siddiqui , and A. S Turk . 2017. “ASPECTS (Alberta Stroke Program Early CT Score) Measurement Using Hounsfield Unit Values When Selecting Patients for Stroke Thrombectomy.” Stroke 48, no. 6: 1574–1579. 10.1161/STROKEAHA.117.016745. 28487329

[brb370144-bib-0023] Nam, K. W. , H. S. Guk , H. M. Kwon , and Y. S. Lee . 2019. “Diffusion‐Weighted Imaging Patterns According to the Right‐to‐Left Shunt Amount in Cryptogenic Stroke.” Cerebrovascular Diseases 48: 45–52. 10.1159/000502882. 31494647

[brb370144-bib-0024] Palazzo, P. , P. Ingrand , P. Agius , R. Belhadj Chaidi , and J. P Neau . 2019. “Transcranial Doppler to Detect Right‐to‐left Shunt in Cryptogenic Acute Ischemic Stroke.” Brain and Behavior 9, no. 1: e01091. 10.1002/brb3.1091. 30506983 PMC6346730

[brb370144-bib-0025] Park, S. , J. K. Oh , J. K. Song , et al. 2021. “Transcranial Doppler as a Screening Tool for High‐Risk Patent Foramen Ovale in Cryptogenic Stroke.” Journal of Neuroimaging 31, no. 1: 165–170. 10.1111/jon.12783. 32896963

[brb370144-bib-0026] Pristipino, C. , H. Sievert , F. D'Ascenzo , et al. 2019. “European Position Paper on the Management of Patients With Patent Foramen Ovale: General Approach and Left Circulation Thromboembolism.” European Heart Journal 40, no. 38: 3182–3195. 10.1093/eurheartj/ehy649. 30358849

[brb370144-bib-0027] Puetz, V. , P. N. Sylaja , S. B. Coutts , et al. 2008. “Extent of Hypoattenuation on CT Angiography Source Images Predicts Functional Outcome in Patients With Basilar Artery Occlusion.” Stroke 39, no. 9: 2486–2490. 10.1161/STROKEAHA.107.511162. 18617663

[brb370144-bib-0028] Robba, C. , and F. S. Taccone . 2019. “How I Use Transcranial Doppler Taccone.” Critical Care 23: 420. 10.1186/s13054-019-2700-6. 31870405 PMC6929281

[brb370144-bib-0029] Rodrigues, A. C. , M. H. Picard , A. Carbone , et al. 2013. “Importance of Adequately Performed Valsalva Maneuver to Detect Patent Foramen Ovale During Transesophageal Echocardiography.” Journal of the American Society of Echocardiography 26: 1337–1343. 10.1016/j.echo.2013.07.016. 23993693

[brb370144-bib-0030] Schneider, B. , T. Zienkiewicz , V. Jansen , T. Hofmann , H. Noltenius , and T. Meinertz . 1996. “Diagnosis of Patent Foramen Ovale by Transesophageal Echocardiography and Correlation With Autopsy Findings.” American Journal of Cardiology 77: 1202–1209. 10.1016/S0002-9149(96)00163-4. 8651096

[brb370144-bib-0031] Seidel, G. , M. Kaps , and T. Gerriets . 1995. “Potential and Limitations of Transcranial Color‐Coded Sonography in Stroke Patients.” Stroke 26, no. 11: 2061–2066. 7482650 10.1161/01.str.26.11.2061

[brb370144-bib-0032] Sloan, M. A. , A. V. Alexandrov , C. H. Tegeler , et al. 2004. “Assessment: Transcranial Doppler Ultrasonography: Report of the Therapeutics and Technology Assessment Subcommittee of the American Academy of Neurology.” Neurology 62: 1468–1481. 10.1212/WNL.62.9.1468. 15136667

[brb370144-bib-0033] Soliman, O. I. , D. A. Theuns , F. J. Ten Cate , et al. 2007. “Predictors of Cardiac Events After Cardiac Resynchronization Therapy With Tissue Doppler‑Derived Parameters.” Journal of Cardiac Failure 13: 805–811. 18068612 10.1016/j.cardfail.2007.07.010

[brb370144-bib-0034] Søndergaard, L. , S. E. Kasner , J. F. Rhodes , et al. 2017. “Patent Foramen Ovale Closure or Antiplatelet Therapy for Cryptogenic Stroke.” New England Journal of Medicine 377: 1033–1042. 10.1056/NEJMoa1707404. 28902580

[brb370144-bib-0035] Spencer, M. P. , M. A. Moehring , J. Jesurum , W. A. Gray , J. V. Olsen , and M. Reisman . 2004. “Power M‐Mode Transcranial Doppler for Diagnosis of Patent Foramen Ovale and Assessing Transcatheter Closure.” Journal of Neuroimaging 14: 342–349. 10.1111/j.1552-6569.2004.tb00261.x. 15358955

[brb370144-bib-0036] Sposato, L. A. , C. S. W. Albin , M. S. V. Elkind , H. Kamel , and J. L. Saver . 2024. “Patent Foramen Ovale Management for Secondary Stroke Prevention: State‐of‐the‐Art Appraisal of Current Evidence.” Stroke 55, no. 1: 236–247. 10.1161/STROKEAHA.123.040546. 38134261

[brb370144-bib-0037] Stecco, A. , M. Quagliozzi , E. Soligo , et al. 2017. “Can Neuroimaging Differentiate PFO and AF‐Related Cardioembolic Stroke From the Other Embolic Sources?” Radiologia Medica 122: 412–418. 10.1007/s11547-017-0738-6. 28224399

[brb370144-bib-0038] Stickland, M. K. , R. C. Welsh , M. J. Haykowsky , et al. 2004. “Intra‐Pulmonary Shunt and Pulmonary Gas Exchange During Exercise in Humans.” Journal of Physiology 561: 321–329. 15388775 10.1113/jphysiol.2004.069302 PMC1665323

[brb370144-bib-0039] Tobe, J. , C. Bogiatzi , C. Munoz , A. Tamayo , and J. D. Spence . 2016. “Transcranial Doppler Is Complementary to Echocardiography for Detection and Risk Stratification of Patent Foramen Ovale.” Canadian Journal of Cardiology 32: 986.e9–986.e16. 10.1016/j.cjca.2015.12.009 26952158

[brb370144-bib-0040] Wessler, B. S. , D. M. Kent , D. E. Thaler , R. Ruthazer , J. S. Lutz , and J. Serena . 2015. “The RoPE Score and Right‐to‐ left Shunt Severity by Transcranial Doppler in the CODICIA Study.” Cerebrovascular Disease 40: 52–58. 10.1159/000430998. PMC4523222 26184495

